# On the Laws Regarding the Mixing of Fluids and Their Penetration into Permeable Substances, with Special Reference to the Processes in the Human and Animal Organism

**Published:** 1847-07

**Authors:** 


### ART. XX.

*Ueber die Gesetze nach welchen die Mischung von Flüssigkeiten und ihr Eindringen in permeable Substanzen erfolgt, mit besonderer Rücksicht auf die Vorgänge im menschlichen und thierischen Organismus.* Von JULIUS VOGEL.—Göttingen, 1846.

*On the Laws regarding the Mixing of Fluids and their penetration into permeable substances, with special reference to the processes in the Human and Animal Organism.* By JULIUS VOGEL.—8vo, pp. 42.

THIS is an excellent attempt to explain many of the obscure points in the process of secretion, by a reference to purely physical laws. It must be obvious to the most casual observer that many phenomena in the human body exclusively depend on the permeation of fluids through organic partitions, whilst in many others it plays a more or less important part. Hence the obvious necessity for a careful investigation of the law regulating the commixture of fluids through membranous partitions, such as occur in the human body.

"The food of which we partake passes first through the mouth, the fauces, and the œsophagus: all these parts are covered by a thick epithelium, which does not readily yield a passage to fluids. In the stomach, however, whose walls permit fluids to pass more readily through them, an energetic reaction takes place between its fluid contents and the blood in the gastric vessels, and a similar process is continued throughout the whole of the intestinal canal. The contents of the stomach are generally much more aqueous than the blood; the various kinds of drink and most articles of fluid food are naturally so, while the saliva and gastric juice reduce solid food to that condition. As is usually the case, where a concentrated fluid comes in contact with one that is more aqueous, there can be no doubt that here also a larger quantity of the thinner fluid passes through the membranous partition to the concentrated fluid, than conversely. Thus by degrees a larger portion of the contents of the stomach and intestinal canal passes into the blood than is conversely yielded by the blood to the fluid of the digestive canal, as has been directly proved by the experiments of Poiseuille. We are still deficient in exact researches respecting the substances that pass from the blood-vessels to the contents of the intestinal canal; but they are probably salts with a small quantity of extractive matters and some protein-compounds, which latter are there converted into mucus. What is usually denominated gastric and intestinal mucus is doubtless the equivalent yielded in the act of digestion by the blood to the contents of the stomach and intestinal canal; but it must be understood that gastric and intestinal mucus may also be secreted by the blood, in the manner we shall subsequently consider under the head of secretions, without that fluid receiving anything in return. It is, however, arranged in a most wonderful manner, that nearly the whole contents of the stomach and intestinal canal may gradually penetrate into the interior of the organism without any appreciable quantity of matter passing as an equivalent from the blood to the intestinal canal. In the first place the acid character of the gastric juice seems to be of great importance. The experiments of Dutrochet show us that acid fluids, especially in their mixture with another fluid through an animal membrane, yield more than they receive; and thus the acid character of the contents of the stomach seems to be precisely the means by which resorption is promoted in a simple physical manner. It would, however, be highly desirable to institute a series of carefully conducted experiments on the influence which the acidity of the gastric juice thus apparently exercises on the resorption of the chyme." (pp. 25-6.)

Vogel then proceeds to illustrate the law, that "the more two fluids separated by an animal membrane differ from one another in the degree of their concentration, the more proportionally will the concentrated abstract from the thinner fluid." This law explains how it is that the passage of nutriment into the circulation is essentially facilitated by the liquidity of the contents of the intestinal canal, induced by the admixture of fluids much more aqueous than the blood, namely, the bile and pancreatic fluid, and further by the drink for which a desire is usually experienced after meal-times.

"The bile contains in itself much more water than the blood, containing on an average about 10 per cent. of solid constituents, while the blood contains more than 20 per cent. The bile, however, becomes yet more aqueous in the intestinal canal owing to a portion of its solid constituents (the bilate of soda) being decomposed by the acid of the chyme, and separated in part in a modified (insoluble) condition, as dyslysin, &c. It contributes therefore essentially to the dilution of the chyme, facilitating its resorption. The pancreatic fluid acts in a like manner, for although we know but little of its composition, it is at all events much more aqueous than the blood, its solid constituents being about 8 per cent. Another means acting with the same object is the circulation of the blood, the consequence of which is, that the portion of blood become more dilute by the absorption of aqueous constituents from the chyme is being constantly removed and replaced by more concentrated blood." (p. 26.)

The object of the lacteals is, according to our author, to abstract the chyle that is too concentrated to be taken up in the above manner, and to take up fatty matter.

"Fat does not mix with water, and as it can either not at all or only with difficulty pass through membranes moistened by watery solutions, it cannot penetrate in more than a very small quantity from the chyme into the blood-vessels. Fat may, however, penetrate from the intestinal canal into the lacteals as has been shown by experiments made on the chyle of animals, killed soon after partaking of fatty food. The penetration of fat through the intestinal walls into the chyle-vessels, is no doubt effected in the same way that oil penetrates through a filter moistened with water; thus in some parts of the filter the particles of oil accumulate so much as to displace the water, the filter becoming saturated with oil at these points, and then forming as it were bridges for the passage of the succeeding oleaginous particles, which are forced into the lacteals by mechanical pressure in the same manner as the aqueous particles of the chyle. Fat, therefore, is not dissolved by the action of the digestive fluid, but the solution is effected by the heat of the body, and fatty mixtures whose fusion-point is higher than 104° F., are either not at all digested (that is to say, resorbed) or only very gradually dissolved by the agency of more fluid fat, subsequently taken into the system by the food. In the resorption of fatty food the surface of the intestinal canal divides itself as it were into two parts, of which one takes up the aqueous chyle and the other the fat. The digestion of fatty food requires a longer period than that of aqueous food, owing to the time required by the fat to force the water from the walls of the separate villi. The resorption of aqueous fluids is rendered more difficult when fat has been previously taken on an empty stomach, for the surface of the intestinal canal becomes invested with a coating of oil which hinders the passage of water. This explains the reason of the inconvenience experienced on partaking copiously of water after eating fatty food, and in like manner why the effect of intoxicating drinks (as beer, for instance) may be retarded for some time by taking a few spoonfuls of oil on an empty stomach." (pp. 28-9.)

Our author then proceeds to explain on similar grounds why it is that the intoxicating effect of a given quantity of alcohol depends in a great measure on the degree of dilution, and to notice the cause of the purgative effect of saline solutions; but as the latter of these views has been prominently brought forward by Liebig and Dr. Golding Bird, we proceed to notice the concluding pages of his pamphlet devoted to the subject of secretion. After stating his views regarding the secretion of sweat, tears, seminal fluid, mucus, and saliva, he gives the following account of the action of the glands of the stomach:

"The gastric glands present certain peculiarities: the product of their secretion so far coincides with the saliva, that it consists of an aqueous fluid with salts and extractive matters, whilst the remains of dissolved gland-cells testify to the presence of protein consumed and modified by organization. But the gastric juice exhibits the peculiarity of containing a free acid. The manner in which this acid is conveyed to it is so much the more difficult of explanation that we do not even clearly know its chemical character, for while it was formerly regarded as hydrochloric acid, it is supposed by more recent investigators to be lactic, combined with a little phosphoric acid; which latter, however, simply owes its origin to the action of the lactic acid upon the alkaline phosphates simultaneously present. By what means is the presence of the free acid brought about? The answer to this question is difficult, and many conjectures may be hazarded on the subject. The acid undoubtedly owes its origin to the blood, and the process of its formation may be owing to the decomposition of some of the salts (alkaline lactates or chlorides) of that fluid by the cells of the gastric glands which retain the acid, while the alkali returns by diffusion into the blood. As the cells gradually become broken up and dissolved, the acid is liberated and mixes with the gastric juice." (pp. 37-8.)

We regret that our limits will not permit us to follow our author through his remarks on the secretion of bile and urine; but we trust we have already stated enough to impress on our readers the importance of the phenomena of the diffusion of fluids within the body; for it is only by a clear understanding of the laws regulating these phenomena and those of molecular chemical actions, that we can ever hope satisfactorily to elucidate the various metamorphoses of matter ever occurring in organised beings.

